# Cryoballoon ablation vs. antiarrhythmic drug therapy as initial treatment for persistent atrial fibrillation: *the multicentre randomized CEPAF trial*

**DOI:** 10.1093/europace/euag230

**Published:** 2026-09-01

**Authors:** Tianyou Ling, Yazhou Lin, Yangyang Bao, Lin Chen, Wei Xu, Baopeng Tang, Jing Xu, Jingquan Zhong, Wei Li, Zhongbao Ruan, Qi Lu, Suxia Guo, Jingfeng Wang, Bing Han, Kai Tang, Tong Liu, Rui Zeng, Jiangui He, Hong Zhi, Changjian Lin, Yue Wei, Zhijun Wu, Ning Zhang, Qi Jin, Yu Yu, Liqun Wu

**Affiliations:** Department of Cardiology, Ruijin Hospital, Shanghai Jiao Tong University School of Medicine, 197 Ruijin Second Road, Shanghai 200025, China; Shengli Clinical Medical College of Fujian Medical University, Fuzhou University Affiliated Provincial Hospital, Fuzhou, China; Department of Cardiology, Ruijin Hospital, Shanghai Jiao Tong University School of Medicine, 197 Ruijin Second Road, Shanghai 200025, China; Shengli Clinical Medical College of Fujian Medical University, Fuzhou University Affiliated Provincial Hospital, Fuzhou, China; Nanjing Drum Tower Hospital, The Affiliated Hospital of Nanjing University Medical School, 321 Zhongshan Road, Nanjing 210008, China; Cardiac Pacing and Electrophysiology Department, Xinjiang Key Laboratory of Cardiac Electrophysiology and Cardiac Remodeling, The First Affiliated Hospital of Xinjiang Medical University, 137 South Liyushan Road, Urumqi 830011, China; Tianjin Chest Hospital, School of Medicine, Tianjin University, Tianjin, China; Department of Cardiology, Qilu Hospital of Shandong University, Jinan, China; Department of Cardiology, Qilu Hospital of Shandong University (Qingdao), Qingdao, China; Department of Cardiovascular Medicine, The Affiliated Hospital of Guizhou Medical University, Guiyang 550004, Guizhou, China; Department of Cardiology, The Affiliated Taizhou People's Hospital of Nanjing Medical University, Taizhou, China; Department of Cardiology, Affiliated Hospital of Nantong University, Nantong 226000, Jiangsu, China; The Tenth Affiliated Hospital of Southern Medical University (Dongguan People's Hospital), Southern Medical University, Dongguan 523018, China; Department of Cardiology, Sun Yat-sen Memorial Hospital, Sun Yat-sen University, Guangzhou, Guangdong 510120, PRC; Department of Cardiology, Xuzhou Central Hospital, Xuzhou, China; Department of Cardiology, Shanghai Tenth People's Hospital, Tongji University, School of Medicine, Shanghai, China; Tianjin Key Laboratory of Ion and Molecular Function of Cardiovascular Diseases, Department of Cardiology, Second Hospital of Tianjin Medical University, Tianjin Institute of Cardiology, Tianjin, China; Department of Cardiology, West China Hospital, Sichuan University, Chengdu, China; Department of Cardiology, The First Affiliated Hospital of Sun Yat-sen University, Guangzhou, China; Department of Cardiology, Zhongda Hospital, Southeast University, Nanjing, China; Department of Cardiology, Ruijin Hospital, Shanghai Jiao Tong University School of Medicine, 197 Ruijin Second Road, Shanghai 200025, China; Department of Cardiology, Ruijin Hospital, Shanghai Jiao Tong University School of Medicine, 197 Ruijin Second Road, Shanghai 200025, China; Department of Cardiology, Ruijin Hospital, Shanghai Jiao Tong University School of Medicine, 197 Ruijin Second Road, Shanghai 200025, China; Department of Cardiology, Ruijin Hospital, Shanghai Jiao Tong University School of Medicine, 197 Ruijin Second Road, Shanghai 200025, China; Department of Cardiology, Ruijin Hospital, Shanghai Jiao Tong University School of Medicine, 197 Ruijin Second Road, Shanghai 200025, China; Department of Cardiology, Ruijin Hospital, Shanghai Jiao Tong University School of Medicine, 197 Ruijin Second Road, Shanghai 200025, China; Department of Cardiology, Ruijin Hospital, Shanghai Jiao Tong University School of Medicine, 197 Ruijin Second Road, Shanghai 200025, China

**Keywords:** Cryoballoon ablation, Antiarrhythmic drug, Persistent atrial fibrillation, Pulmonary vein isolation, Quality of life

## Abstract

**Aims:**

Evidence supporting first-line cryoballoon ablation (CBA) in treatment-naive persistent atrial fibrillation (PersAF) is limited. Consequently, we compared CBA with guideline-directed antiarrhythmic drug (AAD) therapy in this advanced form of disease.

**Methods and results:**

This multicentre, prospective, randomized, open-label trial assigned 320 symptomatic, treatment-naive patients with PersAF (1:1) to CBA or AAD therapy. The primary endpoint was the first documented atrial tachyarrhythmia recurrence lasting at least 30 s during days 91–365. Secondary endpoints included recurrence during days 1–90 and 1–365, time-to-first recurrence, quality of life, and safety. In the intention-to-treat population, recurrence during days 91–365 occurred in 30/160 (18.8%) patients assigned to CBA and 71/160 (44.4%) assigned to AAD therapy (absolute difference, −25.6 percentage points; 95% confidence interval, −35.4 to −15.8; *P* < 0.001). Supportive time-to-event analysis favoured CBA (hazard ratio for AAD vs. CBA, 2.29; 95% confidence interval, 1.48–3.56; log-rank *P* < 0.001). In CBA vs. AAD comparisons, overall recurrence during days 1–365 was 23.8% vs. 55.6%, and early recurrence was 11.2% vs. 28.1% (both *P* < 0.001; respectively). Improvement in AFEQT score was greater with CBA (17.3 vs. 6.6 points; *P* < 0.001), whereas SF-12 component scores did not differ significantly. Adverse events recorded on event forms were infrequent in both cohorts.

**Conclusion:**

In selected treatment-naive patients with PersAF, first-line CBA reduced atrial tachyarrhythmia recurrence and improved disease-specific quality of life compared with AAD therapy.

**Trial Registration:**

ClinicalTrials.gov, NCT04942834.

## Graphical Abstract

**Graphical Abstract:**
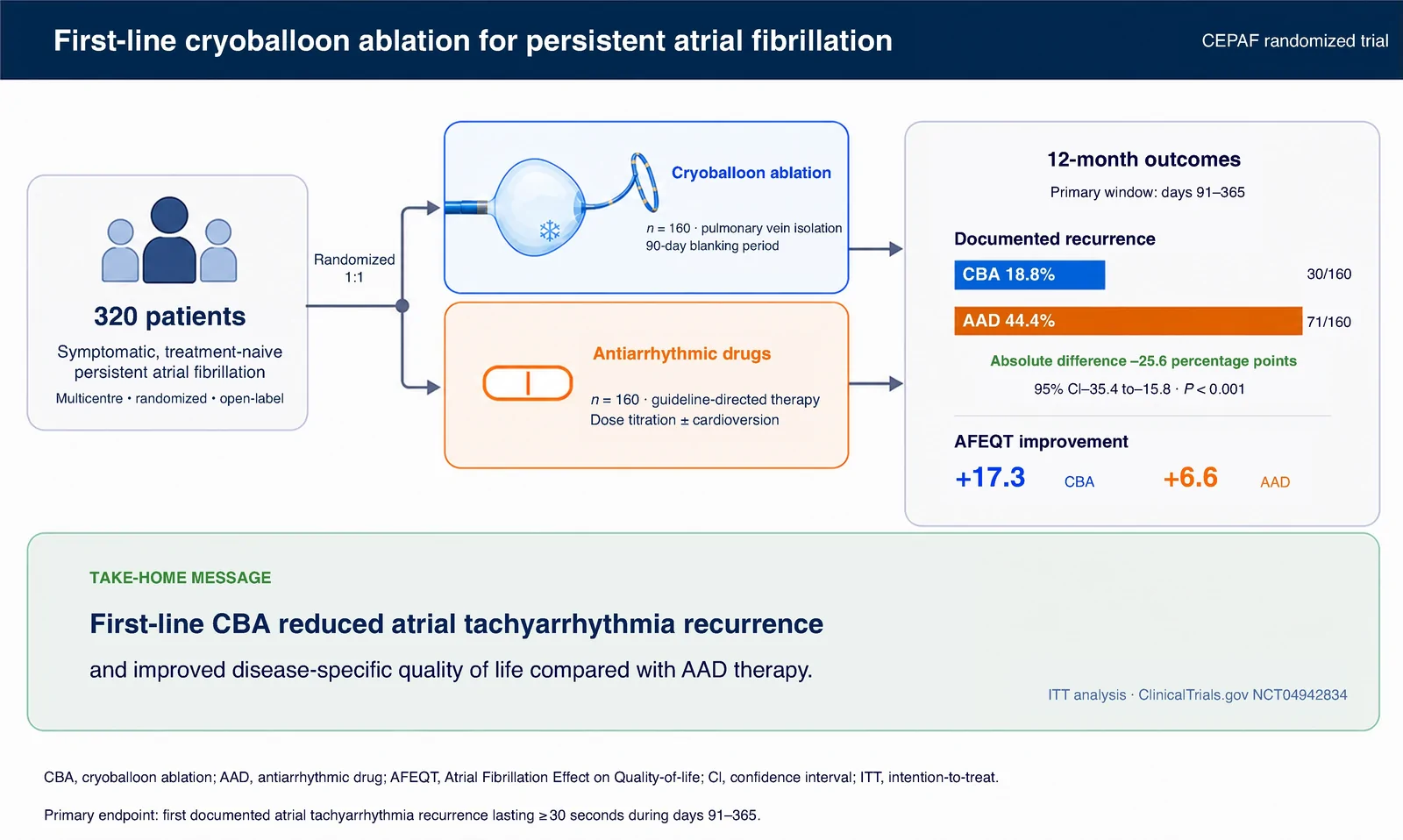

What’s new?

- In this multicentre randomized trial of 320 treatment-naive patients with persistent atrial fibrillation, first-line cryoballoon ablation reduced documented atrial tachyarrhythmia recurrence during days 91–365 compared with antiarrhythmic drug therapy (18.8% vs. 44.4%).
- Cryoballoon ablation also reduced recurrence over the full 12-month follow-up and produced a greater improvement in disease-specific quality of life.
- These findings extend randomized evidence for initial catheter ablation to selected patients with persistent atrial fibrillation without advanced structural heart disease.

## Background

Atrial fibrillation (AF) is the most common sustained cardiac arrhythmia and remains a major contributor to global morbidity. Recent Global Burden of Disease analyses have shown that the worldwide burden of atrial fibrillation and atrial flutter continued to increase from 1990 to 2021, with important sex- and region-specific differences and projected future growth.^1,2^ AF is significantly associated with stroke, heart failure, and mortality risks, which severely increase the healthcare and social burden. Among AF phenotypes, persistent AF (PersAF) poses an enhanced challenge for rhythm control due to its longer duration and pronounced structural remodelling.^3,4^

In patients with drug-refractory AF, catheter ablation is more effective than AAD therapy for maintaining sinus rhythm,^5,6^ and multicentre studies of CBA in PersAF have reported 12-month single-procedure success rates of 60.3–69.0%.^7,8^ Although rhythm control remains a cornerstone of management for PersAF, guideline recommendations regarding catheter ablation as initial therapy are not uniform.^5,9^ The evolving role of catheter ablation in contemporary rhythm-control strategies is further discussed in the Europace Spotlight within the 2024 ESC/EACTS guidelines.^10^ This uncertainty (in early intervention strategy) is clinically relevant because AAD-based rhythm control has only modest efficacy in PersAF, with reported success rates of 30–50%.^11–13^ Additionally, prolonged AF may promote atrial remodelling and reduce the likelihood of successful subsequent rhythm-control interventions.^14,15^ Nevertheless, randomized evidence directly comparing initial CBA with AAD therapy in treatment-naive patients with PersAF remains limited. Thus, the CEPAF trial compared CBA with AAD therapy as initial rhythm-control strategies in treatment-naive patients with symptomatic PersAF.

## Methods

### Study registration and ethics

CEPAF was a researcher-initiated, prospective, multicentre, open-label randomized trial comparing CBA with guideline-directed AAD therapy as an initial rhythm-control treatment in patients with symptomatic, treatment-naive PersAF. The trial was prospectively registered at ClinicalTrials.gov (NCT04942834). Each participating centre obtained ethics approval, and all participants provided written informed consent. The study was conducted in accordance with the Declaration of Helsinki and applicable regulations. An independent Data Safety Monitoring Board oversaw safety, and an independent Event Adjudication Committee applied common evidence standards for event reviews while blinded to treatment assignment (details in the Supplementary material online, Appendix).

### Study design

The study used a parallel-group design with 1:1 randomization via centralized assignments and enrolled 320 participants (160 per group) at 17 centres in China between 2021 and 2025. Enrolment, treatment, and follow-up were conducted according to the detailed study protocol provided in the Supplementary material online, Appendix.

### Study patients

Adults aged 18 to 75 years with symptomatic PersAF lasting more than 7 days and less than 1 year were eligible for study enrolment. Key exclusion criteria were previous left atrial ablation or surgery; prior use of class I or III antiarrhythmic drugs for AF for more than 7 consecutive days; substantial structural heart disease (left ventricular ejection fraction <50%, interventricular septal thickness >12 mm, left atrial anteroposterior diameter >46 mm, or moderate-to-severe valvular disease) or QRS duration >120 ms; pulmonary vein stenosis or prior pulmonary vein stenting; prior phrenic-nerve paralysis; implanted devices that could compromise rhythm ascertainment; active infection; contraindications to anticoagulation or left atrial thrombus; acute coronary syndrome, percutaneous coronary intervention, or coronary-artery bypass grafting within 3 months; New York Heart Association class III or IV heart failure; estimated glomerular filtration rate <30 mL per minute per 1.73 m^2^; pregnancy or breastfeeding; life expectancy <1 year; or anticipated poor adherence to study follow-up. Detailed information is provided in the Supplementary material (Study Protocol).

### Randomization and blinding

Participants were randomized using a centrally generated, stratified block randomization scheme, stratified by centre with permuted blocks within each centre (with 1:1 allocation). The randomization scheme (including block sizes) was prespecified and retained by an independent statistician, and allocation concealment was ensured using a sealed, opaque, sequentially numbered envelope system prepared centrally. After written informed consent was obtained and eligibility confirmed, the next envelope in sequence at the enrolling centre was opened to assign participants to CBA or AAD therapy. The trial was an open-label design; however, rhythm and safety outcomes were adjudicated in a blinded manner. Specifically, all Holter and electrocardiogram (ECG) recordings were interpreted by a central core laboratory, and clinical events were adjudicated by an independent Event Adjudication Committee whose members were blinded to treatment assignments.

### Cryoballoon ablation (CBA)

Before ablation, anticoagulation was prescribed according to guideline recommendations. All participants received at least 3 weeks of therapeutic oral anticoagulation or underwent transoesophageal echocardiography to exclude left atrial thrombus before the procedure. A single transseptal puncture via femoral vein access was obtained with conscious sedation or general anaesthesia selected based on the centre’s standard of practice. Unfractionated heparin was administered to maintain ACT between 250 and 350 s, and pulmonary vein isolation (entrance/exit block) was the acute endpoint at the ablation conclusion. The ablation devices used included the Arctic Front Advance 23- or 28-mm (paired with the Achieve mapping catheter). Intraoperatively, metrics such as occlusion grading, time to isolation (TTI), number/duration of freezes, and minimum temperature were recorded. Routine phrenic nerve pacing and real-time monitoring were performed during right pulmonary vein ablations. Continuous perioperative anticoagulation (e.g. VKA/DOAC) was used. If AF or atrial tachycardia persisted at the end of the procedure, electrical cardioversion was performed to restore sinus rhythm and to allow for confirmation of durable bidirectional conduction block of all targeted pulmonary veins. The postoperative blanking period lasted from 0 to 90 days, and it allowed for short-term use of AAD and/or electrical cardioversion during this period without efficacy failure. Furthermore, AADs were discontinued after 90 days if no recurrence(s) were observed, as defined in the study protocol.

### Antiarrhythmic drugs (AAD)

Patients allocated to the AAD arm received ‘guideline-directed’ rhythm-control therapy during a prespecified 0 to 90-day treatment period (blanking period), with dose titration and cardioversion as needed. In patients without significant structural heart disease, class Ic therapy (propafenone) was preferred when clinically appropriate. Propafenone was initiated at 150 mg two or three times daily and combined with atrioventricular nodal blockade (e.g. a beta-blocker or non-dihydropyridine calcium channel blocker) when appropriate. Class III options included dronedarone at 400 mg twice daily and sotalol at 40–80 mg twice daily (dose-adjusted for renal function and QT interval monitoring). Amiodarone was not preferred as a routine ‘first-line’ maintenance therapy because of extracardiac toxicity, but it was permitted when other AADs were unsuitable or ineffective and for clinically indicated rhythm stabilization. By prespecified trial design, any requirement for escalation of class I/III AAD therapy to maintain sinus rhythm (beyond day 90) was adjudicated as a treatment failure (in sensitivity analyses).

### Endpoints and definitions

The primary efficacy endpoint was defined as the first occurrence of ≥30 s of atrial arrhythmia (AF/atrial flutter/atrial tachycardia) between days 91 and 365 after treatment initiation (with objective electrocardiographic evidence). Secondary efficacy endpoints were denoted as the early recurrence of atrial arrhythmias (days 1–90); overall recurrence of atrial arrhythmias (days 1–365); time-to-first recurrence of atrial arrhythmias (days 91–365 and days 1–365); repeat ablation procedures between days 91–365; and quality-of-life changes (via AFEQT and SF-12) from baseline to 12 months.

### Safety Endpoints

Adverse events were reported from treatment initiation through 12 months, and all events were accounted using an adverse event(s) form. Prespecified adverse events included gastrointestinal bleeding and new clinically significant arrhythmias. Additionally, serious adverse outcomes included death, myocardial infarction, stroke, systemic embolism, and heart failure. All safety events were adjudicated in a blinded manner by an independent Event Adjudication Committee based on source documents.

### Follow-up and rhythm monitoring

Baseline evaluation included medical history, physical examination, 12-lead ECG, echocardiography, and laboratory tests. Transesophageal echocardiography or ≥3 weeks of therapeutic oral anticoagulation was used when needed to exclude left atrial thrombus. Participants in both groups were scheduled for follow-up visits at 3 months (visit window ±15 days), 6 months (±15 days), and 12 months (±30 days) after treatment initiation. A 12-lead ECG was obtained at each visit, and Holter monitoring was performed at 3, 6, and 12 months and when symptoms occurred. If AF persisted at the 3-month visit, electrical cardioversion was recommended. All ECG and Holter data were centrally interpreted by the core laboratory.

### Statistical analysis

The primary efficacy analysis included all randomized participants according to assigned treatment group. Categorical variables are presented as counts (percentages), and continuous variables are denoted as means (SDs) or medians (interquartile ranges), as appropriate. All statistical tests were two-sided, with a significance threshold of *P* < 0.05, and effect estimates are reported with 95% confidence intervals (CIs). For the primary analysis, documented recurrences were counted as events. The per-protocol analysis is reported in Supplementary material online, *Table S1*. Sensitivity analyses classified missing primary endpoints as recurrence and counted post-blanking class I/III AAD usage after CBA as ‘treatment failure’ (see Supplementary material online, *Table S2*).

‘Time-to-event’ outcomes were summarized using Kaplan-Meier methods as supportive analyses. Treatment effects were estimated using Cox proportional hazards models with treatment group as the primary covariate and are reported as hazard ratios (HRs) with 95% CIs. Participants with missing primary endpoint information were retained in the ITT population, and when no documented recurrence was available, follow-up was censored at the last available rhythm assessment rather than excluding the participant from the randomized denominator.

Multiple imputations by chained equations were used for missing baseline covariates and missing quality-of-life endpoints. Fifty imputed datasets were generated (using treatment group, age, BMI, sex, hypertension, diabetes, vascular disease, documented recurrence indicators, and available quality-of-life measures), and estimates were combined using Rubin's rules. All analyses were performed using SPSS version 29.0.1.0 and R version 4.2.2.

## Results

### Patients

**Figure 1:**
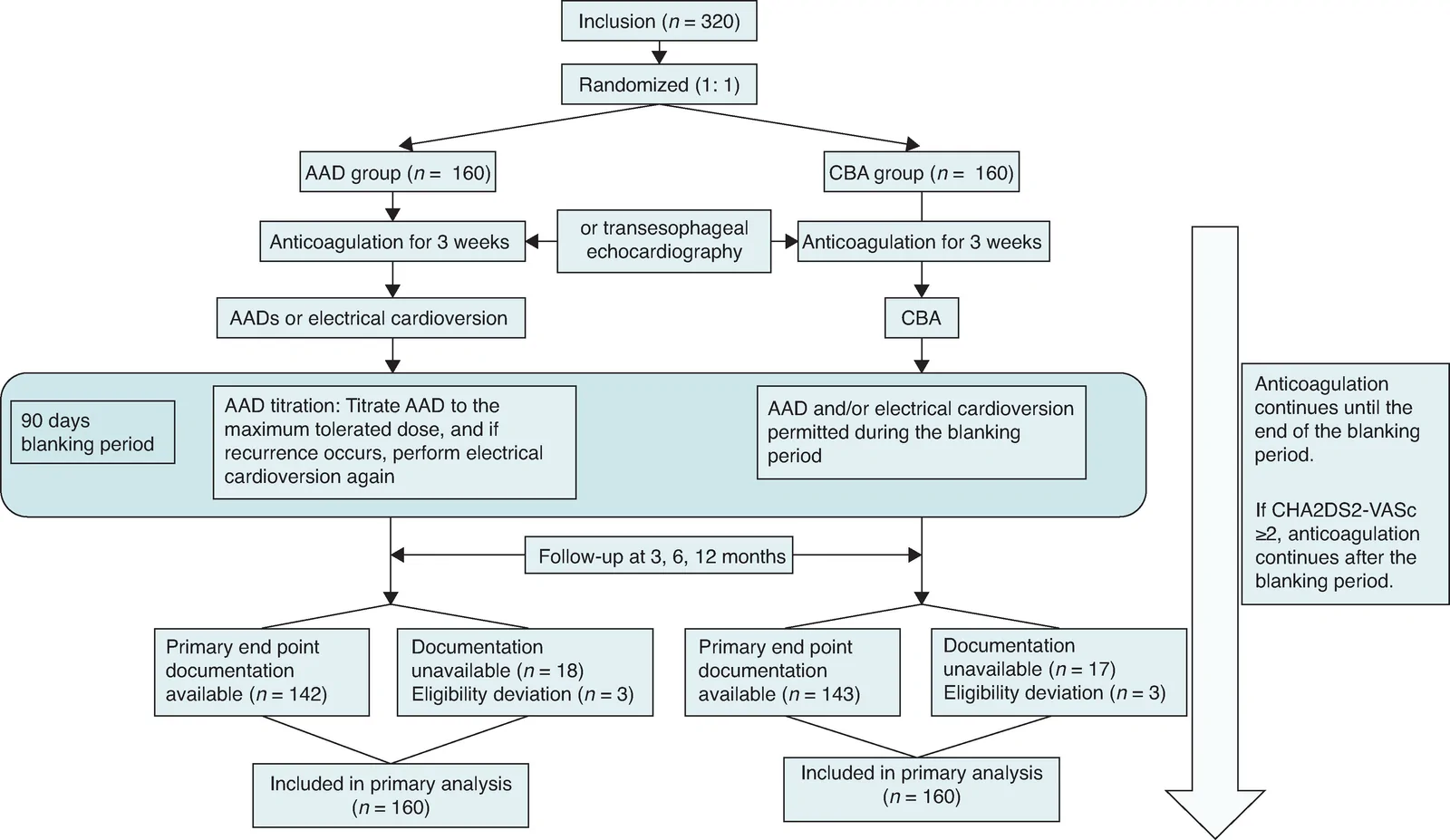
Participant flow diagram. Abbreviations: AAD, antiarrhythmic drug; CBA, cryoballoon ablation.

A total of 320 participants were enrolled and randomized (160 to CBA and 160 to AAD), and all randomized participants were retained in the primary analysis. All participants in the CBA group underwent CBA, and all participants in the AAD group received AAD therapy. After randomization, three participants in each group were found to ‘not satisfy’ an eligibility criterion; however, all six remained in their assigned groups for the primary analysis. Primary endpoint documentation was unavailable for 17 participants assigned to CBA and 18 assigned to AAD therapy. Regarding CBA vs. AAD, the last structured follow-up record was absent for 10 and 13 participants (respectively), and ‘last follow-up’ occurred at 3 months for 1 and 4 participants, at 6 months for 3 and 0 participants, and at 9 months for 3 and 1 participants, respectively. All 35 participants remained in their randomized groups, and the effects (of missing outcome data) were examined in sensitivity analyses (*Figure 1* and Supplementary material online, *Table S3*).

Baseline characteristics for the randomized population are shown in *Table 1*. The groups were broadly similar, although BMI and recorded anticoagulant/antiplatelet usage showed descriptive imbalances. In accordance with CONSORT guidance, no significance tests are presented for randomized baseline comparisons.

**Table 1:** Characteristics of the patients at baseline

Characteristic	Overall	Cryoballoon ablation	AAD therapy
Age, y	61.4 ± 9.9	61.0 ± 9.9	61.8 ± 9.9
Male sex, no. (%)	226 (70.6)	109 (68.1)	117 (73.1)
Female sex, no. (%)	94 (29.4)	51 (31.9)	43 (26.9)
Body mass index, kg/m^2^	24.8 ± 3.1	25.5 ± 3.2	24.0 ± 2.9
Current smoker, no. (%)	23 (7.2)	13 (8.1)	10 (6.2)
NYHA class I or II, no. (%)	72 (22.5)	40 (25.0)	32 (20.0)
Hypertension, no. (%)	150 (46.9)	83 (51.9)	67 (41.9)
Ventricular arrhythmia, no. (%)	8 (2.5)	2 (1.2)	6 (3.8)
Vascular disease, no. (%)	39 (12.2)	18 (11.2)	21 (13.1)
Diabetes, no. (%)	29 (9.1)	19 (11.9)	10 (6.2)
CHA_2_DS_2_-VASc score	1.7 ± 1.3	1.8 ± 1.3	1.6 ± 1.3
Baseline amiodarone, no. (%)	18 (5.6)	11 (6.9)	7 (4.4)
Baseline propafenone, no. (%)	11 (3.4)	8 (5.0)	3 (1.9)
Baseline sotalol, no. (%)	2 (0.6)	1 (0.6)	1 (0.6)
Any anticoagulant/antiplatelet recorded, no. (%)	151 (47.2)	86 (53.8)	65 (40.6)
QRS duration, ms	102.5 ± 15.7	101.5 ± 15.3	103.6 ± 16.1
QTc interval, ms	424.6 ± 22.8	421.9 ± 24.2	427.1 ± 21.2
Left atrial diameter, mm	41.3 ± 4.6	41.1 ± 4.7	41.5 ± 4.5
Left ventricular ejection fraction, %	61.1 ± 6.3	61.5 ± 6.3	60.8 ± 6.3
Interventricular septum thickness, mm	9.5 ± 1.2	9.6 ± 1.2	9.5 ± 1.3
AFEQT score	66.6 ± 17.8	65.6 ± 17.7	67.7 ± 17.9
SF-12 PCS baseline	91.0 ± 25.9	91.0 ± 27.0	91.0 ± 24.8
SF-12 MCS baseline	67.0 ± 20.8	67.7 ± 21.7	66.3 ± 19.9

Values are mean ± SD or *n* (%), unless otherwise indicated. Continuous summaries are based on available data. In accordance with CONSORT guidance, significance tests are not presented for randomized baseline comparisons.

### Primary endpoint

#### First recurrence between days 91 and 365

**Figure 2:**
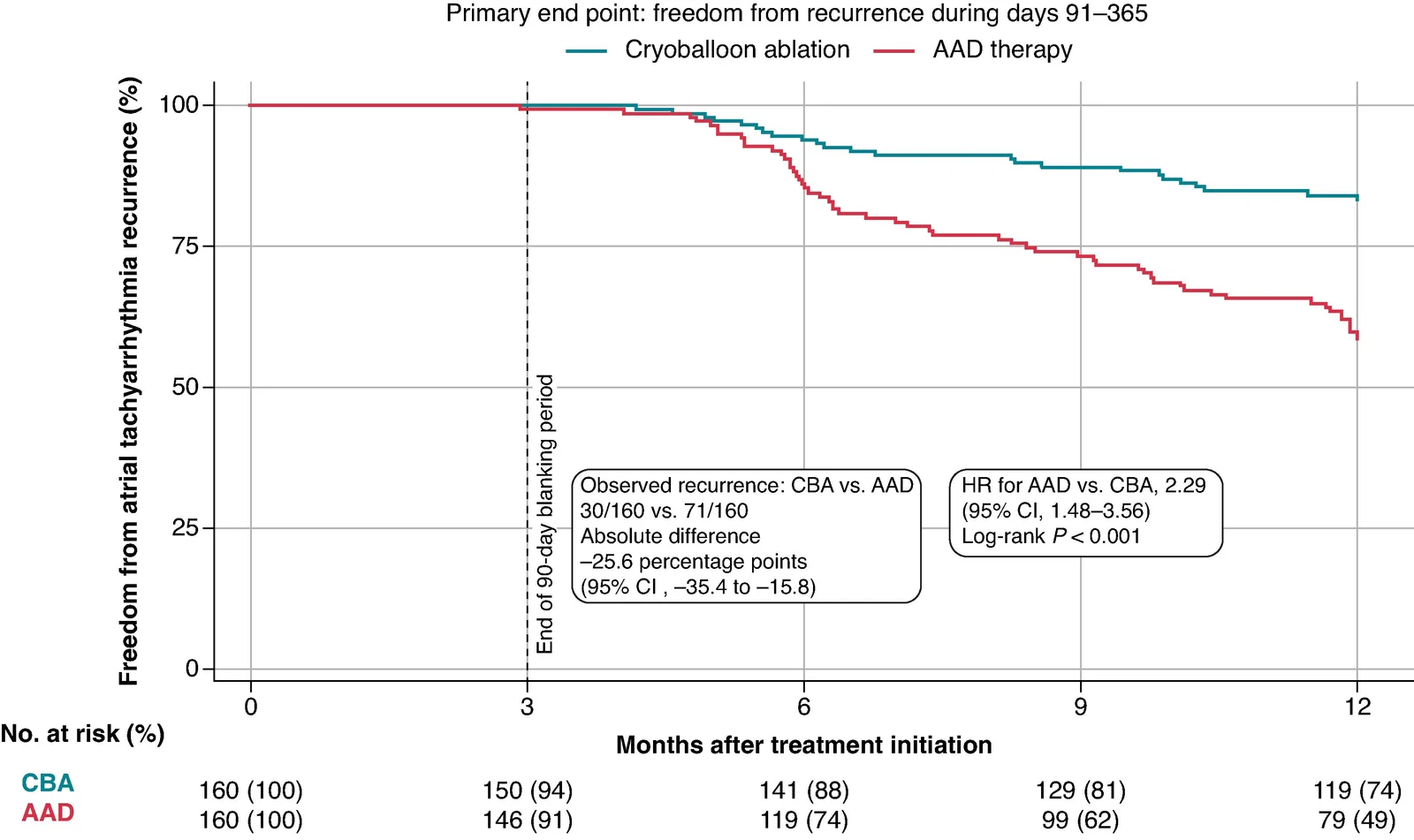
Freedom from atrial tachyarrhythmia recurrence during days 91–365 (primary endpoint). Kaplan-Meier estimate shows freedom from first documented atrial arrhythmia recurrence during days 91–365 after treatment initiation in the ITT population. Participants without documented recurrence were retained in the ITT cohort and censored at the last available rhythm follow-up. The Cox model HR for AAD therapy vs. CBA was 2.29 (95% CI, 1.48 to 3.56; log-rank *P* < 0.001). Numbers at risk are shown below. The dashed line marks the end of the blanking period.

During the primary evaluation window, documented recurrence occurred in 30/160 (18.8%) in the CBA group and 71/160 (44.4%) in the AAD group in the ITT primary analysis, with an absolute difference of −25.6 percentage points (95% CI, −35.4 to −15.8; *P* < 0.001) (*Table 2*). The supportive Kaplan-Meier analysis retained all randomized participants and censored participants without documented recurrence at the last available rhythm follow-up, and the HR for AAD therapy vs. CBA was 2.29 (95% CI, 1.48 to 3.56; log-rank *P* < 0.001) (*Figure 2*).

**Table 2:** Primary and secondary endpoints

Endpoint	Cryoballoon ablation (*n* = 160)	AAD therapy (*n* = 160)	Difference (95% CI)	*P* value
Recurrence of atrial tachyarrhythmia, no. (%)				
Days 91 to 365: primary ITT endpoint	30 (18.8)	71 (44.4)	−25.6 (−35.4, −15.8)	<0.001
Days 1 to 90	18 (11.2)	45 (28.1)	−16.9 (−25.4, −8.4)	<0.001
Days 1 to 365	38 (23.8)	89 (55.6)	−31.9 (−42.0, −21.7)	<0.001
Additional ablation between days 91 and 365, no. (%)	3 (1.9)	20 (12.5)	−10.6 (−16.2, −5.1)	<0.001
No. of days since first recurrence of atrial arrhythmia				
Within days 91 to 365	264.9 ± 104.9	282.8 ± 117.6	−17.9 (−65.0, 29.2)	0.456
Within days 1 to 365	179.1 ± 146.8	185.6 ± 140.0	−6.6 (−62.1, 49.0)	0.817
Quality-of-life assessments				
AFEQT score at 12 mo	83.1 ± 11.0	74.3 ± 15.4	8.8 (5.0, 12.5)	<0.001
SF-12 PCS at 12 mo	96.3 ± 22.4	96.1 ± 23.8	0.3 (−5.5, 6.1)	0.929
SF-12 MCS at 12 mo	79.2 ± 20.6	80.5 ± 19.1	−1.3 (−6.3, 3.6)	0.601
Change from baseline to 12 mo in AFEQT score	17.3 ± 17.3	6.6 ± 19.9	10.6 (5.2, 16.1)	<0.001
Change from baseline to 12 mo in SF-12 PCS score	5.3 ± 34.0	5.0 ± 33.2	0.3 (−7.7, 8.3)	0.941
Change from baseline to 12 mo in SF-12 MCS score	11.5 ± 27.9	14.0 ± 26.4	−2.6 (−9.1, 4.0)	0.445

Analyses are based on the intention-to-treat set. SF-12 estimates were pooled across 50 chained-equation imputations using Rubin's rules.

### Secondary endpoints

#### Recurrence during the blanking period (1–90 days)

CBA (11.2%; 18/160) vs. AAD (28.1%; 45/160) demonstrated significantly lower atrial arrhythmia recurrence in the CBA cohort during the blanking period, with an absolute difference of −16.9 percentage points (95% CI, −25.4 to −8.4; *P* < 0.001) (*Table 2*).

#### Recurrence over the year (1–365 days)

**Figure 3:**
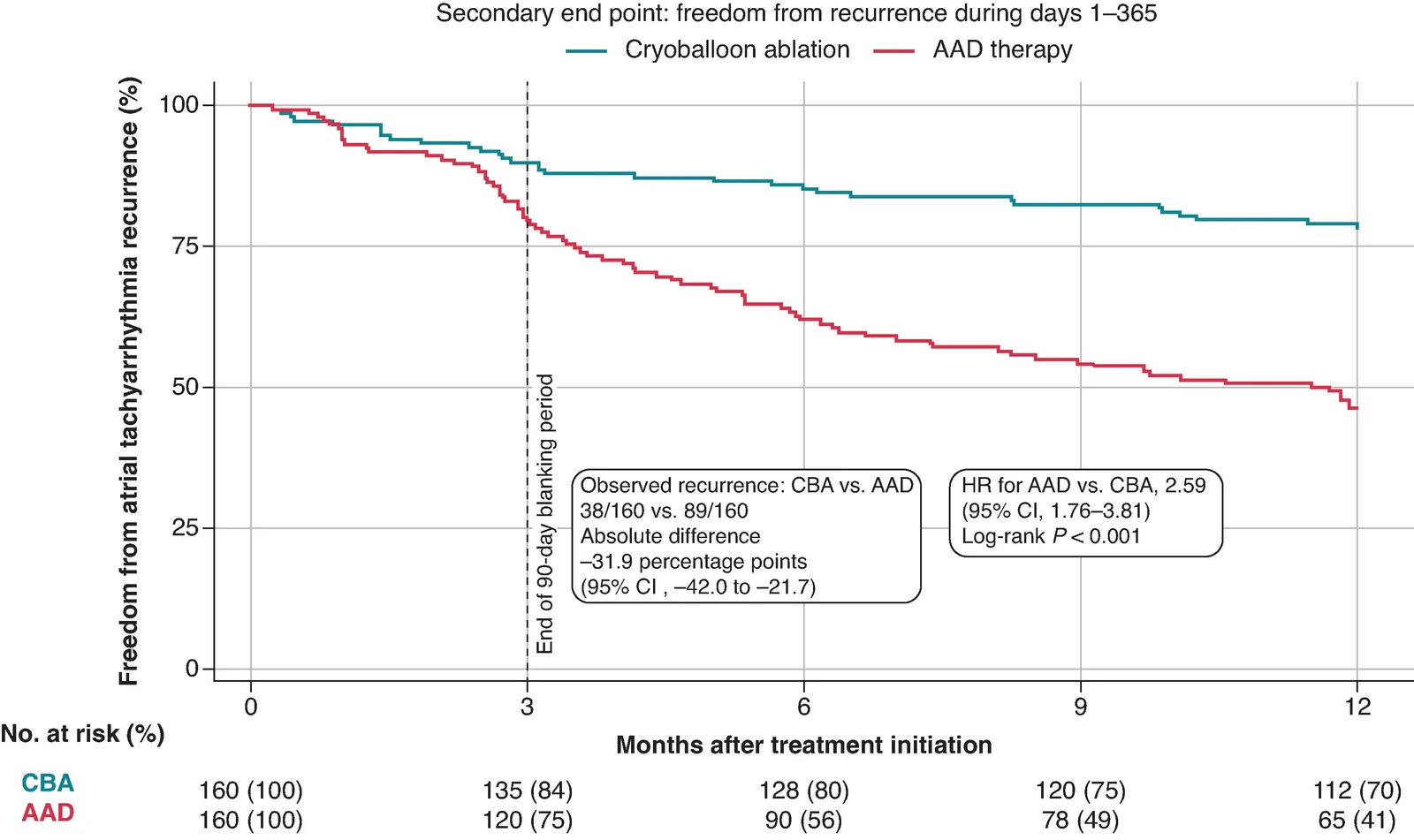
Freedom from atrial tachyarrhythmia recurrence from day 1 to day 365. Kaplan-Meier estimate show freedom from first documented recurrence from day 1 through day 365 after treatment initiation in the ITT population. Participants without documented recurrence were retained in the ITT cohort and censored at the last available rhythm follow-up. The Cox model HR for AAD therapy vs. CBA was 2.59 (95% CI, 1.76 to 3.81; log-rank *P* < 0.001). Numbers at risk are shown below.

CBA (23.8%; 38/160) vs. AAD (55.6%; 89/160) demonstrated significantly lower atrial arrhythmia recurrence in the CBA cohort during the entire trial follow-up period, with an absolute difference of −31.9 percentage points (95% CI, −42.0 to −21.7; *P* < 0.001) (*Table 2*). The supportive time-to-event analysis also favoured CBA (HR for AAD therapy vs. CBA, 2.59; 95% CI, 1.76 to 3.81; log-rank *P* < 0.001) (*Figure 3*).

#### Time-to-first recurrence

In the days 91–365 window, the time-to-first recurrence among participants with observed recurrence was 264.9 ± 104.9 days in the CBA arm and 282.8 ± 117.6 days in the AAD arm (difference, −17.9 days; 95% CI, −65.0 to 29.2; *P* = 0.456). In the days 1–365 window, it was 179.1 ± 146.8 days in the CBA arm and 185.6 ± 140.0 days in the AAD arm (difference, −6.6 days; 95% CI, −62.1 to 49.0; *P* = 0.817) (*Table 2*).

#### Quality of life

At 12 months, the AFEQT score was higher in the CBA group than in the AAD group (83.1 ± 11.0 vs. 74.3 ± 15.4 with a ‘between-group’ difference of 8.8 [95% CI, 5.0 to 12.5]; *P* < 0.001). Change from baseline to 12 months in AFEQT score was positive in both groups and favoured CBA (17.3 ± 17.3 vs. 6.6 ± 19.9; between-group difference of 10.6 [95% CI, 5.2 to 16.1]; *P* < 0.001). After a 50-fold multiple imputation, the 12-month PCS score was 96.3 ± 22.4 with CBA and 96.1 ± 23.8 with AAD (difference of 0.3 [95% CI, −5.5 to 6.1]; *P* = 0.929), and the 12-month MCS score was 79.2 ± 20.6 and 80.5 ± 19.1, respectively (difference of −1.3 [95% CI, −6.3 to 3.6]; *P* = 0.601). Changes in PCS and MCS did not differ significantly between groups (*Table 2* and Supplementary material online, *Table S4*).

#### Safety

Adverse events recorded on AE forms were infrequent (*Table 3* and Supplementary material online, *Table S5*), and any (AE form) denoted event occurred in 0/160 participants in the CBA group and 3/160 participants in the AAD group. No deaths, myocardial infarctions, strokes/embolisms, hospitalizations due to heart failure, or persistent phrenic-nerve palsy were recorded.

**Table 3:** Adverse events

Event, no. (%)	Overall (*n* = 320)	Cryoballoon ablation (*n* = 160)	AAD therapy (*n* = 160)
Any adverse event recorded on AE form	3 (0.9)	0 (0.0)	3 (1.9)
Serious adverse event	2 (0.6)	0 (0.0)	2 (1.2)
Gastrointestinal bleeding	1 (0.3)	0 (0.0)	1 (0.6)
Other arrhythmias	1 (0.3)	0 (0.0)	1 (0.6)
Chest discomfort	1 (0.3)	0 (0.0)	1 (0.6)
Death	0 (0.0)	0 (0.0)	0 (0.0)
Myocardial infarction	0 (0.0)	0 (0.0)	0 (0.0)
Stroke	0 (0.0)	0 (0.0)	0 (0.0)
Embolic event	0 (0.0)	0 (0.0)	0 (0.0)
Heart failure	0 (0.0)	0 (0.0)	0 (0.0)
Persistent phrenic-nerve palsy	0 (0.0)	0 (0.0)	NA

Data are presented as *n* (%).

AE, adverse event; AAD, antiarrhythmic drug; NA, not applicable.

#### Treatment characteristics

Supplementary material online, *Table S6* summarizes antiarrhythmic drug therapy recorded in the AAD arm at the 3-month visit (corresponding to the end of the blanking period). Overall, 68/160 AAD-arm participants had one of the listed class I/III AAD records, including amiodarone 44/160 (27.5%), propafenone 12/160 (7.5%), sotalol 6/160 (3.8%), dronedarone 4/160 (2.5%), and moricizine 2/160 (1.2%), with condensed dose information.

#### Sensitivity analysis

Sensitivity analyses yielded results consistent with the primary analysis. In the conservative analysis in which participants with missing primary endpoint data were classified as having recurrence, recurrence between days 91 and 365 remained lower with CBA than with AAD therapy (47/160 [29.4%] vs. 89/160 [55.6%]; Supplementary material online, *Table S2*). In the treatment-failure sensitivity analysis that counted CBA participants continuing class I/III AAD therapy after blanking as failures, the result also remained significant (33/160 [20.6%] vs. 71/160 [44.4%] when missing endpoints were censored, and 50/160 [31.2%] vs. 89/160 [55.6%] when missing endpoints were classified as recurrence; Supplementary material online, *Table S2*). The per-protocol analysis was also consistent with the primary result (20.7% vs. 51.1%; Supplementary material online, *Table S1*).

Given the baseline imbalance in BMI, we performed an additional sensitivity Cox analysis with administrative censoring at day 365 and multiple imputation of missing BMI. In this analysis, the treatment effect remained significant after BMI adjustment (HR for AAD therapy vs. CBA, 2.61; 95% CI, 1.68 to 4.04; *P* < 0.001), consistent with the unadjusted estimate obtained using the same event-time rule (HR, 2.76; 95% CI, 1.80 to 4.22; *P* < 0.001) (see Supplementary material online, *Table S7*).

## Discussion

In this randomized trial, first-line CBA reduced documented atrial tachyarrhythmia recurrence compared with AAD therapy among treatment-naive patients with PersAF and limited structural heart disease. The difference was present during the primary endpoint window (days 91–365) and across the full 12-month follow-up. CBA also produced a greater improvement in disease-specific quality of life, whereas SF-12 component scores were similar between groups. Additionally, both treatments were safe with a low adverse event rate in each cohort.

Our findings are consistent with the long-term rhythm-control advantage observed in the CABANA trial.^16^ Also, the PRAGUE-25 trial found better rhythm outcomes with ablation than with lifestyle modification plus AAD therapy in patients with obesity.^17^ These studies and CEPAF support catheter ablation as an effective rhythm-control option for persistent AF, while differences in study populations and comparators should be considered.

Previous first-line CBA trials (Cryo-FIRST, STOP AF First, and EARLY-AF) focused on younger, healthier cohorts with paroxysmal AF.^18–20^ CEPAF extends this evidence to selected patients with persistent AF, a population with a more extensive arrhythmic substrate and a greater burden of cardiovascular comorbidity. The recently reported AVANT GUARD trial provides complementary evidence for first-line ablation in persistent AF,^21^ and the trial’s usage of continuous rhythm monitoring strengthened the outcome ascertainment.

CEPAF used a standardized PVI-only strategy, consistent with the STOP Persistent AF and PRECEPT studies.^8,22^ These studies showed that PVI-based ablation can provide clinically meaningful rhythm control in persistent AF. In congruence, STAR AF II found no additional benefit from empiric linear ablation or complex fractionated atrial electrogram ablation, and the CAPLA study did not support routine posterior wall isolation.^3,4^ Consequently, the standardized PVI-only strategy used in CEPAF provides a clear comparison with initial drug therapy without the confounding factor of ablation strategy (used in the trial).

The trial enrolled treatment-naive patients with persistent AF who did not have advanced structural heart disease. This selection allowed the comparison to address intervention timing before substantial atrial remodelling or treatment failure. The findings are consistent with EAST-AFNET 4, which showed clinical benefit from early rhythm control,^23^ and add randomized evidence for an initial CBA strategy in a selected persistent AF population. Given the variation in current guideline recommendations regarding first-line ablation for persistent AF,^5,9^ our findings provide additional randomized evidence to inform the choice between initial cryoballoon ablation and antiarrhythmic drug therapy. Importantly, the results of the CEPAF trial agree with previous studies that support early rhythm control benefit(s) via catheter ablation, and now, these observations of patient benefit are extended to included individuals with more advanced forms of atrial fibrillation.

## Limitations

Our trial has several limitations. First, the 12-month follow-up may underestimate late recurrence and long-term complications. Second, primary endpoint documentation was unavailable for 35 participants (10.9%). Although these participants remained in the randomized comparison and conservative sensitivity analyses produced similar results; the potential bias from missing data cannot be excluded. Third, rhythm surveillance relied mainly on scheduled and symptom-triggered ECG and Holter monitoring rather than continuous monitoring, so asymptomatic recurrences may have been missed and under reported. Fourth, despite a prespecified protocol and centralized adjudication, residual between-centre and operator-related variability in procedural execution and peri-procedural management may have influenced treatment implementation and outcome ascertainment (which may potentially limit generalizability). Finally, the results require confirmation in populations with different healthcare settings, ethnic backgrounds, obesity profiles, and comorbidity burdens.

## Conclusions

In selected treatment-naive patients with PersAF, first-line CBA reduced atrial tachyarrhythmia recurrence over 12 months and improved disease-specific quality of life compared with AAD therapy. However, longer-term follow-up and continuous rhythm monitoring are needed to define the durability and absolute magnitude of this benefit.

## Supplementary Material

euag230_Supplementary_Data

## Data Availability

The de-identified data underlying this article will be shared upon reasonable request to the corresponding author, subject to applicable ethical, privacy, and institutional requirements.
