# Supplementary material for: Cryoballoon ablation vs. antiarrhythmic drug therapy as initial treatment for persistent atrial fibrillation: the multicentre randomized CEPAF trial
Source: Europace. 2026 Sep 1;28(9):euag230. doi: 10.1093/europace/euag230 (PMC13582022; doi:10.1093/europace/euag230)
Supplement: Supplementary file 1 [file euag230_supplementary_data.docx]

**Supplementary Appendix**

**Detailed CEPAF Trial Protocol and Supplementary Tables**

*ClinicalTrials.gov registration: NCT04942834*

**Detailed Trial Protocol**

**1. Protocol identification and study objective**

CEPAF was a researcher-initiated, prospective, multicentre, open-label, parallel-group randomized trial conducted at 17 centres in China. The trial compared cryoballoon ablation (CBA) with guideline-directed antiarrhythmic drug (AAD) therapy as initial rhythm-control treatment in symptomatic, treatment-naive patients with persistent atrial fibrillation (PersAF). The primary objective was to determine whether CBA reduced documented atrial tachyarrhythmia recurrence during days 91-365 after treatment initiation.

The protocol's primary objective was to compare 12-month freedom from recurrent atrial tachyarrhythmia after a 90-day blanking period. The sample-size calculation assumed 12-month success rates of 55% with CBA and 35% with AAD therapy, a two-sided alpha of 0.05, more than 90% power, and 20% attrition. Planned enrolment was 320 participants, with 160 assigned to each group. The trial was registered at ClinicalTrials.gov (NCT04942834).

**2. Study population**

Inclusion criteria

- Age 18-75 years.
- Symptomatic PersAF documented for more than 7 days and less than 1 year.
- No previous rhythm-control treatment with a class I or III AAD for more than 7 consecutive days.
- Ability to provide written informed consent and comply with scheduled treatment and follow-up.

Exclusion criteria

- Previous left atrial catheter ablation or cardiac surgery involving the left atrium.
- Left ventricular ejection fraction below 50%, interventricular septal thickness above 12 mm, left atrial anteroposterior diameter above 46 mm, or moderate-to-severe valvular disease.
- QRS duration above 120 ms.
- Pulmonary vein stenosis, previous pulmonary vein stenting, or previous phrenic-nerve paralysis.
- A cardiac rhythm device that could compromise protocol-defined rhythm ascertainment.
- Left atrial thrombus, contraindication to anticoagulation, or active systemic infection.
- Acute coronary syndrome, percutaneous coronary intervention, or coronary-artery bypass grafting within 3 months.
- New York Heart Association class III or IV heart failure.
- Estimated glomerular filtration rate below 30 mL/min/1.73 m².
- Pregnancy, breastfeeding, life expectancy below 1 year, or anticipated inability to complete study procedures.

**3. Randomization, allocation concealment, and outcome assessment**

Participants were assigned 1:1 using a centrally generated, centre-stratified block randomization scheme with permuted blocks. Block sizes and the random seed were specified before enrolment and retained by an independent statistician. Allocation was concealed using centrally prepared, sequentially numbered, opaque sealed envelopes. The next envelope was opened after written consent and eligibility confirmation. Treatment was open-label. ECG and Holter recordings were interpreted centrally, and rhythm and safety events were adjudicated by a committee blinded to treatment assignment.

**4. Cryoballoon ablation intervention**

Before ablation, participants received at least 3 weeks of therapeutic oral anticoagulation or underwent transoesophageal echocardiography to exclude left atrial thrombus. CBA was performed through femoral venous access after a single transseptal puncture under conscious sedation or general anaesthesia according to centre practice. Unfractionated heparin was administered to maintain an activated clotting time of 250-350 s. Pulmonary vein isolation was performed with a 23-mm or 28-mm Arctic Front Advance cryoballoon and an Achieve mapping catheter. Pulmonary vein entrance and exit block was required at the end of the procedure. Operators recorded balloon occlusion grade, time to isolation, number and duration of applications, and minimum temperature. The right phrenic nerve was paced and diaphragmatic function monitored during right-sided applications. If AF or atrial tachycardia persisted after ablation, electrical cardioversion was performed. Short-term AAD therapy and cardioversion were permitted during the 90-day blanking period; AAD therapy was stopped after day 90 in the absence of recurrence.

**5. Antiarrhythmic drug intervention**

Participants assigned to AAD therapy received a class Ic or class III agent selected according to clinical characteristics, contraindications, renal function, QT interval, and guideline recommendations. Propafenone was generally started at 150 mg two or three times daily and was combined with atrioventricular nodal blockade when appropriate. Dronedarone was prescribed at 400 mg twice daily. Sotalol was prescribed at 40-80 mg twice daily with renal dose adjustment and QT monitoring. Amiodarone was not preferred for routine first-line maintenance because of extracardiac toxicity but was permitted when other agents were unsuitable or ineffective or when rhythm stabilization was clinically required. Dose titration and electrical cardioversion were allowed during the initial 90-day treatment period. Drug and dose records at the end of the blanking period are summarized in Supplementary Table S6.

**6. Anticoagulation and cardioversion**

Anticoagulation was prescribed according to guideline recommendations. Therapeutic anticoagulation was continued through the blanking period. Continuation after day 90 was based on thromboembolic risk and clinical indications. Electrical cardioversion was permitted when needed to restore sinus rhythm; cardioversion was recommended when AF persisted at the 3-month visit.

**7. Follow-up and rhythm surveillance**

Participants were scheduled for visits at 3 months (window ±15 days), 6 months (±15 days), and 12 months (±30 days) after treatment initiation. A 12-lead ECG was obtained at each scheduled visit. Holter monitoring was planned at 3, 6, and 12 months and whenever symptoms suggested recurrence. All available ECG and Holter recordings were assessed by the central core laboratory.

**Protocol Table 1. Schedule of assessments.**

| **Assessment** | **Baseline** | **Treatment/day 0** | **3 months** | **6 months** | **12 months** | **Unscheduled** |
| --- | --- | --- | --- | --- | --- | --- |
| Eligibility and consent | Yes | - | - | - | - | - |
| Medical history/physical examination | Yes | As needed | Yes | Yes | Yes | As needed |
| 12-lead ECG | Yes | Yes | Yes | Yes | Yes | If symptomatic |
| Holter monitoring | Baseline rhythm assessment | As needed | Yes | Yes | Yes | If symptomatic |
| Echocardiography | Yes | As clinically indicated | - | - | As clinically indicated | - |
| Medication review | Yes | Yes | Yes | Yes | Yes | As needed |
| AFEQT and SF-12 | Yes | - | - | - | Yes | - |
| Adverse-event assessment | Baseline review | Yes | Yes | Yes | Yes | Yes |

*AFEQT, Atrial Fibrillation Effect on Quality-of-Life questionnaire; ECG, electrocardiogram; SF-12, 12-Item Short Form Health Survey.*

**8. End points**

**Primary efficacy end point.** The first objectively documented episode of AF, atrial flutter, or atrial tachycardia lasting at least 30 s during days 91-365 after treatment initiation.

**Secondary efficacy end points.** Documented recurrence during days 1-90 and days 1-365; time to first recurrence during days 91-365 and days 1-365; repeat ablation during days 91-365; and change in AFEQT and SF-12 scores from baseline to 12 months.

**Safety end points.** Adverse events recorded on trial adverse-event forms, including serious events and prespecified outcomes of death, myocardial infarction, stroke or systemic embolism, heart failure, and persistent phrenic-nerve palsy. Serious ablation-related safety end points. Transient ischaemic attack, cerebrovascular accident, major bleeding requiring transfusion, cardiac perforation/tamponade/pericardial effusion, myocardial infarction, or death within 7 days; pulmonary vein stenosis with at least 75% luminal reduction within 12 months; phrenic-nerve injury unresolved at 12 months; and atrio-oesophageal fistula within 12 months.

**9. Analysis populations and statistical methods**

The primary efficacy analysis included all randomized participants according to assigned treatment group. Binary end points are reported as counts, percentages, absolute risk differences, 95% confidence intervals, and two-sided P values. Kaplan-Meier curves and Cox proportional-hazards models were used as supportive time-to-event analyses. Participants without documented recurrence remained in the randomized denominator and were censored at the last rhythm assessment in time-to-event analyses. Sensitivity analyses classified missing primary end points as recurrence and counted continued class I/III AAD therapy after CBA beyond day 90 as treatment failure. A per-protocol analysis was retained as a supplementary sensitivity analysis. Missing baseline covariates and quality-of-life outcomes were addressed with 50-fold multiple imputation by chained equations and Rubin pooling.

**10. Data quality, oversight, and ethics**

Source documents and case-report forms were reviewed at participating centres. ECG and Holter recordings were interpreted by a central core laboratory. An independent Event Adjudication Committee, blinded to treatment assignment, adjudicated rhythm and safety events using common evidence standards. Ethics approval was obtained at each participating centre, all participants provided written informed consent, and the study was conducted in accordance with the Declaration of Helsinki.

**Supplementary Tables**

**Supplementary Table S1. Per-protocol analysis of primary and secondary end points.**

| **End point** | **CBA (n=135)** | **AAD therapy (n=139)** | **Difference (95% CI)** | **P value** |
| --- | --- | --- | --- | --- |
| Days 91-365: primary end point | 28 (20.7) | 71 (51.1) | -30.3 (-41.1, -19.6) | <0.001 |
| Days 1-90 | 18 (13.3) | 44 (31.7) | -18.3 (-27.9, -8.7) | <0.001 |
| Days 1-365 | 36 (26.7) | 88 (63.3) | -36.6 (-47.6, -25.7) | <0.001 |

*Values are n (%). CBA, cryoballoon ablation; CI, confidence interval.*

**Supplementary Table S2. Sensitivity analyses for the primary end point.**

| **Analysis** | **CBA (n=160)** | **AAD therapy (n=160)** | **Difference (95% CI)** | **P value** |
| --- | --- | --- | --- | --- |
| Documented recurrence; randomized denominator | 30 (18.8) | 71 (44.4) | -25.6 (-35.4, -15.8) | <0.001 |
| Missing primary end point classified as recurrence | 47 (29.4) | 89 (55.6) | -26.2 (-36.7, -15.8) | <0.001 |
| Post-blanking AAD after CBA classified as failure | 33 (20.6) | 71 (44.4) | -23.8 (-33.7, -13.8) | <0.001 |
| Post-blanking AAD after CBA classified as failure; missing classified as recurrence | 50 (31.2) | 89 (55.6) | -24.4 (-34.9, -13.8) | <0.001 |

*Values are n (%). Participants remained in their randomized groups. Continued class I/III AAD therapy after CBA beyond day 90 was classified as treatment failure in the indicated analyses.*

**Supplementary Table S3. Participant status and availability of primary end point documentation.**

| **Participant or follow-up status** | **CBA (n=160)** | **AAD therapy (n=160)** |
| --- | --- | --- |
| Eligibility criterion not satisfied after randomization; retained in assigned group | 3 (1.9) | 3 (1.9) |
| Primary end point documentation available | 143 (89.4) | 142 (88.8) |
| Primary end point documentation unavailable | 17 (10.6) | 18 (11.2) |
| No structured post-baseline follow-up record | 10 | 13 |
| Last structured record at 3 months | 1 | 4 |
| Last structured record at 6 months | 3 | 0 |
| Last structured record at 9 months | 3 | 1 |
| Possible outside-hospital recurrence reported without adjudicable ECG documentation | 0 | 1 |

*Values are n (%) for the first three rows and n thereafter. Last-record categories sum to the number with unavailable primary end point documentation. They describe data availability, not reasons for non-completion. All participants remained in the randomized analysis.*

**Supplementary Table S4. SF-12 multiple-imputation estimates.**

| **Outcome** | **CBA** | **AAD therapy** | **Difference (95% CI)** | **P value** |
| --- | --- | --- | --- | --- |
| PCS at 12 months | 96.3 ± 22.4 | 96.1 ± 23.8 | 0.3 (-5.5, 6.1) | 0.929 |
| MCS at 12 months | 79.2 ± 20.6 | 80.5 ± 19.1 | -1.3 (-6.3, 3.6) | 0.601 |
| PCS change | 5.3 ± 34.0 | 5.0 ± 33.2 | 0.3 (-7.7, 8.3) | 0.941 |
| MCS change | 11.5 ± 27.9 | 14.0 ± 26.4 | -2.6 (-9.1, 4.0) | 0.445 |

*Estimates were pooled across 50 chained-equation imputations using Rubin's rules. MCS, mental component summary; PCS, physical component summary; SF-12, 12-Item Short Form Health Survey.*

**Supplementary Table S5. Safety events and adjudication.**

| **Safety item** | **Definition/ascertainment** | **Overall** | **CBA** | **AAD** |
| --- | --- | --- | --- | --- |
| Any AE-form event | At least one event recorded within 12 months | 3/320 | 0/160 | 3/160 |
| Serious adverse event | Seriousness classification on AE form | 2/320 | 0/160 | 2/160 |
| Gastrointestinal bleeding | Adjudicated gastrointestinal bleeding | 1/320 | 0/160 | 1/160 |
| Other arrhythmia | Clinically significant arrhythmia other than recurrent atrial tachyarrhythmia | 1/320 | 0/160 | 1/160 |
| Chest discomfort | AE-form chest discomfort | 1/320 | 0/160 | 1/160 |
| Death/MI/stroke/embolism/heart failure/persistent phrenic palsy | Prespecified serious safety outcomes | 0 | 0 | 0 |

**Supplementary Table S6. Antiarrhythmic drug therapy recorded in the AAD arm at the end of the blanking period.**

| **Visit** | **Drug** | **Patients/160 (%)** | **Condensed dose information from source records** |
| --- | --- | --- | --- |
| 3-month visit | Amiodarone | 44/160 (27.5) | Predominantly oral 100-200 mg qd/qod/bid/tid, including loading-to-maintenance schedules |
| 3-month visit | Propafenone | 12/160 (7.5) | Mostly 150 mg tid; other entries included 200 mg tid, 200 mg qd, and 450 mg qd |
| 3-month visit | Sotalol | 6/160 (3.8) | 40-80 mg qd or 80-120 mg bid, including one 80+40 mg bid entry |
| 3-month visit | Dronedarone | 4/160 (2.5) | 400 mg qd or bid |
| 3-month visit | Moricizine | 2/160 (1.2) | 100 mg bid or 150 mg tid |

*Each row represents unique participants with that drug recorded at the 3-month visit; 68/160 participants had one of the listed class I/III AAD records. These data describe the AAD arm only. bid, twice daily; qd, once daily; qod, every other day; tid, three times daily.*

**Supplementary Table S7. BMI-adjusted sensitivity Cox analysis for recurrence during days 91-365.**

| **Model** | **HR for AAD vs. CBA** | **95% CI** | **P value** |
| --- | --- | --- | --- |
| Unadjusted, same day-365 event-time rule | 2.76 | 1.80-4.22 | <0.001 |
| Adjusted for BMI after 50-fold multiple imputation | 2.61 | 1.68-4.04 | <0.001 |

*The documented primary-event classification was used. Exact recorded event times within days 91-365 were used; documented events with missing or out-of-window times were assigned to day 365, and participants without documented recurrence were administratively censored at day 365. BMI, body mass index; CI, confidence interval; HR, hazard ratio.*
